# Detecting Pharmacovigilance Signals Combining Electronic Medical Records With Spontaneous Reports: A Case Study of Conventional Disease-Modifying Antirheumatic Drugs for Rheumatoid Arthritis

**DOI:** 10.3389/fphar.2018.00875

**Published:** 2018-08-07

**Authors:** Liwei Wang, Majid Rastegar-Mojarad, Zhiliang Ji, Sijia Liu, Ke Liu, Sungrim Moon, Feichen Shen, Yanshan Wang, Lixia Yao, John M. Davis III, Hongfang Liu

**Affiliations:** ^1^Department of Health Sciences Research, Mayo Clinic College of Medicine, Rochester, MN, United States; ^2^State Key Laboratory of Cellular Stress Biology, School of Life Sciences, Xiamen University, Xiamen, China

**Keywords:** adverse drug event, disease-modifying antirheumatic drug (DMARD), Electronic Medical Records (EMR), FDA Adverse Event Reporting System (FAERS), pharmacovigilance, natural language processing

## Abstract

Multiple data sources are preferred in adverse drug event (ADEs) surveillance owing to inadequacies of single source. However, analytic methods to monitor potential ADEs after prolonged drug exposure are still lacking. In this study we propose a method aiming to screen potential ADEs by combining FDA Adverse Event Reporting System (FAERS) and Electronic Medical Record (EMR). The proposed method uses natural language processing (NLP) techniques to extract treatment outcome information captured in unstructured text and adopts case-crossover design in EMR. Performances were evaluated using two ADE knowledge bases: Adverse Drug Reaction Classification System (ADReCS) and SIDER. We tested our method in ADE signal detection of conventional disease-modifying antirheumatic drugs (DMARDs) in rheumatoid arthritis patients. Findings showed that recall greatly increased when combining FAERS with EMR compared with FAERS alone and EMR alone, especially for flexible mapping strategy. Precision (FAERS + EMR) in detecting ADEs improved using ADReCS as gold standard compared with SIDER. In addition, signals detected from EMR have considerably overlapped with signals detected from FAERS or ADE knowledge bases, implying the importance of EMR for pharmacovigilance. ADE signals detected from EMR and/or FAERS but not in existing knowledge bases provide hypothesis for future study.

## Introduction

Adverse drug event (ADE), the untoward occurrence after exposure to a drug (Pirmohamed et al., 1998), has been an important public health concern. It is well recognized that pre-marketing randomized controlled trials (RCTs) may not detect all types of ADEs related to a particular drug in clinical practice (Schneeweiss and Avorn, 2005) due to some limitations (Stolberg et al., 2004). Therefore, FDA encourages the public to voluntarily report any suspicious ADEs to facilitate pharmacovigilance. The FDA Adverse Event Reporting System (FAERS) has become an important resource in post-marketing surveillance for ADEs (Yang et al., 2011; Jiang et al., 2013; Wang et al., 2014, 2017a; Yu et al., 2016; European Medicines Agency, 2017). Commonly used methods to detect ADE signals from FAERS include Proportional Reporting Ratio (PRR), Reporting Odds Ratio (ROR), Information Component (IC) and Empirical Bayesian Geometric Mean (EBGM) (Bate and Evans, 2009). For example, a recent study discovered the higher risk of glioblastoma associated with tumor necrosis factor inhibitor by using ROR in FAERS (Guo et al., 2016). Since FAERS is a voluntary reporting system, some inherited disadvantages exist such as underreporting and no incidence of ADEs (Stephenson and Hauben, 2007). Other data sources have been studied to assist ADE detection further. Besides social media (Liu and Chen, 2015) and literature (Xu and Wang, 2014), Electronic Medical Record (EMR) has also been paid a lot of attention. E.g., Observational Health Data Sciences and Informatics (OHDSI) has been implementing the international collaboration to conduct clinical outcome research including ADE surveillance using EMR (Hripcsak et al., 2015). A majority of the valuable information about clinical outcomes and medication in EMR is captured in clinical narratives (Classen et al., 2011). To analyze the clinical narratives, natural language processing (NLP) methods have been employed to extract potential drug-ADE pairs. Signal detection methods such as χ^2^ test or odds ratio have been utilized to rank the pairs (Wang et al., 2009, 2010; Harpaz et al., 2013). The feasibility of using EMR to validate potential drug-ADE pairs detected from FAERS has been demonstrated (Wang et al., 2017b). However, a majority of the existing studies used data-driven disproportionality analysis with little consideration of confounding variables such as race and gender. Analytic methods to monitor potential ADEs of a drug after prolonged drug exposure are still lacking (Schuemie et al., 2016). In this study, we propose a method aiming to screen potential ADEs from both FAERS and EMR. Specifically, this method incorporates NLP techniques to process EMR, where case-crossover study is employed with consideration of clinical context, including drug indication and temporal information. Subsequently, the potential ADEs from clinical notes and FAERS are evaluated by comparing against SIDER (Kuhn et al., 2015) and Adverse Drug Reaction Classification System (ADReCS) (Cai et al., 2015). We test our proposed method for screening potential ADEs for conventional disease-modifying antirheumatic drugs (DMARDs), the cornerstone treatment for Rheumatoid arthritis (RA) patients.

## Materials and Methods

**FIGURE 1:**
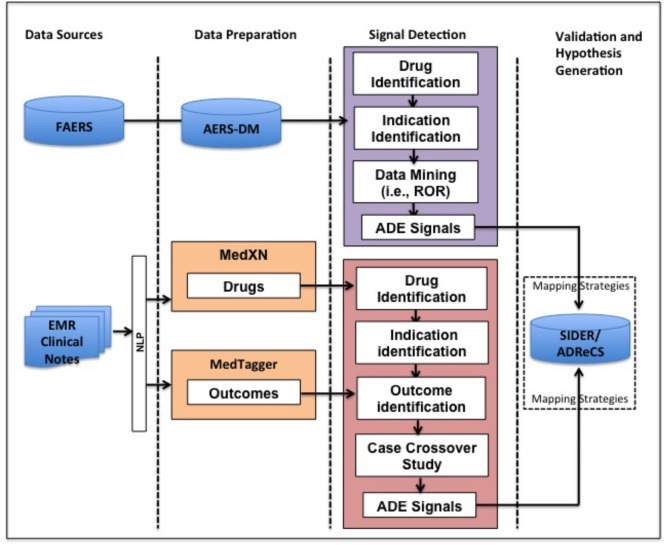
Overview of our method for ADE mining from FAERS and EMR. FAERS, FDA Adverse Event Reporting System; EMR, Electronic Medical Record; ADE, Adverse Drug Event; ROR, Reporting Odds Ratio.

Our method for detecting potential ADEs of a drug and indication pair (D, IND) is featured by NLP techniques and case-crossover study design. Specifically it includes three steps: data preparation, signal detection, and validation and hypothesis generation (**Figure 1**). In the data preparation step, we apply NLP techniques to extract drugs and clinical outcomes, i.e., problems from EMR. In the signal detection step, a cohort of patients taking drug D for IND is identified from longitudinal EMR data and the corresponding spontaneous reports for the pair (D, IND) in FAERS are extracted. Then, we use case-crossover study design to detect potential ADE signals from EMR. Potential ADE signals from FAERS are detected using ROR. In the validation and hypothesis generation step, signals from EMR and FAERS are then normalized and evaluated using ADE knowledge bases, i.e., ADReCS and SIDER where exact and flexible mapping strategies are used. Existing records of ADEs in the knowledge bases serve as the gold standards and false positives (i.e., signals not present in the knowledge bases) are potential ADEs for further investigation, i.e., hypothesis. The study has been approved as a minimal risk study with Mayo IRB of 13-009317. Only the data of patients with research authorization for using their EMR records have been used.

### Data Preparation

In this study we used the normalized knowledge-enhanced data set AERS-DM (Wang et al., 2014) derived from FAERS that is downloadable at our website^1^. The details and summarization for the data processing can be found in our previous paper (Yang et al., 2011; Wang et al., 2017b).

The EMR data used in our study consists of all patient records at Mayo Clinic Rochester campus with Minnesota research permission over a period of 20 years (January 1995–October 2017). As a majority of the clinical information in EMR is captured in unstructured clinical notes, we process clinical notes using MedXN (Sohn et al., 2014), which extracts and normalizes medication mentions to RxNorm and MedTagger (Torii et al., 2011), which extracts and normalizes clinical concept mentions to the Unified Medical Language System (UMLS). We limit our analysis to the following UMLS semantic types: “Finding(T033),” “Laboratory or Test Result(T034),” “Sign or Symptom(T184),” “Disease or Syndrome(T047),” “Mental or Behavioral Dysfunction (T048),” “Neoplastic Process(T191)” and “Cell or Molecular Dysfunction(T049),” which are related to indications and clinical outcomes.

There are two ADE knowledge bases used in our study. One is SIDER, a resource that aggregates dispersed public information on side effects recorded in public documents and package inserts (Kuhn et al., 2015). In the downloadable files, drugs are coded using STITCH compound identifiers, adverse events and indications are coded using both UMLS and MedDRA lowest level term (LLT). We used SIDER 4.1 in our study.

The other is ADReCS, a more comprehensive ADE resource integrating multiple public medical repositories such as SIDER, DailyMed and ClinicalTrials (Cai et al., 2015). ADReCS adopts WHO-ART, MedDRA, and the UMLS as major references for standardization. In this study, ADReCS 1.6 was used.

### Signal Detection From AERS-DM

The associated records of drug D and indication IND are extracted. ROR are computed based on a 2 × 2 contingency table (Evans et al., 2001; Rothman et al., 2004). More specifically the number of reports associated with the drug and ADE is defined as *a*. The number of reports with the drug and without ADE is defined as *b*. The number of reports with other drugs and with ADE is defined as *c*. The number of reports with other drugs and without ADE is defined as *d*. In this analysis, a signal is detected if the lower bound of 95% confidence interval of ROR exceeds 1 (van Puijenbroek et al., 2002). The analysis is implemented in R package PhViD 1.0.6 (Ahmed and Poncet, 2013).

1 $$ROR=\frac{a\times d}{b\times c}$$

### Signal Detection From EMR

We use case-crossover design to detect signals of association between drug D and potential ADEs for patients with IND from EMR (Maclure and Mittleman, 2000). The case-crossover study is a popular epidemiological study design for which individuals act as their own controls, i.e., comparisons are made within individuals. It has been recommended for pharmacoepidemiological research to leverage EMR data as confounding factors, such as genetics and gender, are well controlled and they do not vary with time (Hallas and Pottegård, 2014).

In this study we conducted unconditional analysis using odds ratio (OR) to detect signals. A potential ADE is detected when the lower bound of the 95% confidence interval of the OR exceeds 1 and *p*-value is less than 0.05.

2 $$OR=\frac{a\times d}{b\times c}$$

**FIGURE 2:**
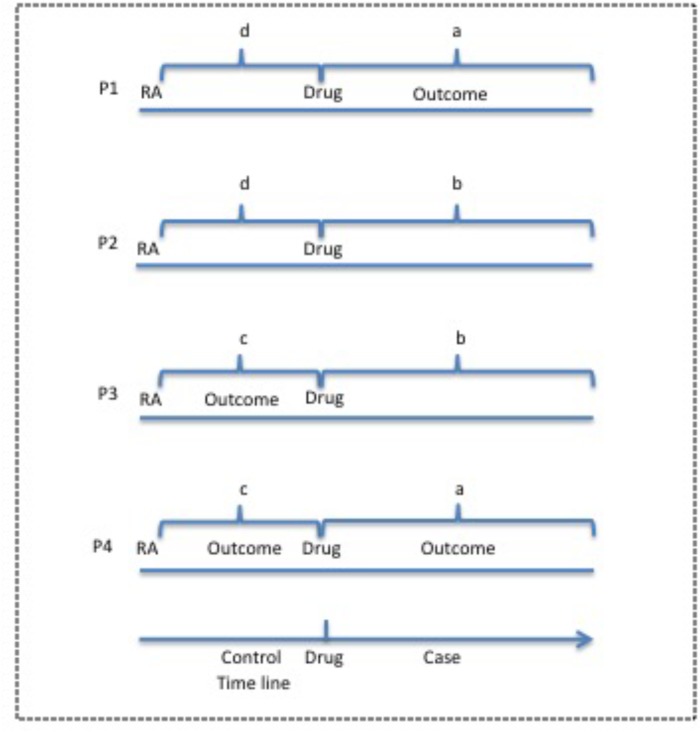
Case-crossover study design for potential ADEs of drugs of interest from EMR clinical notes. The observation period before the use of drug is used as the control and the period after drug use is used as the case. P, Patient; RA, rheumatoid arthritis.

**Figure 2** defines *a*, *b*, *c*, and *d* for OR calculation in case-crossover study. We use the patient cohort during the observation period before the use of drug D and after the diagnosis of IND as the control, and the same patient cohort during the observation period after the first use until the last use of drugs of interest as the case. In the case group, if an outcome occurs, the patient is counted as “a”, otherwise as “b”. In the control group, if an outcome occurs, then the patient is counted as “c”, otherwise as “d”. OR value could be calculated for each outcome using the same patient cohort undergoing drug therapy. Therefore, the patient number in the case group is the same as that in the control group for each outcome.

### Validation and Hypothesis Generation

**FIGURE 3:**
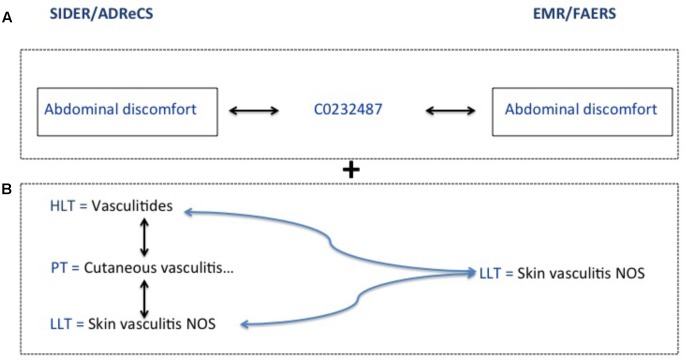
Exact and flexible mapping strategies. **(A)** Refers to exact mapping. **(B)** Refers to flexible mapping.

The ADE obtained from FAERS, EMR, SIDER and ADReCS are coded differently. In order to evaluate the performance of this method for ADE signal detection, we use two mapping strategies to map signals among various data sources. The first strategy is exact mapping. We unify all the codes from various data sources to the UMLS and then conduct direct mapping among sources. The second strategy is flexible mapping combining both exact mapping and the mapping through hierarchical structure. The latter is conducted by leveraging the 5-level hierarchical structure of Medical Dictionary for Regulatory Activities (MedDRA) (Bousquet et al., 2005). Specifically, we expand high-level terms of MedDRA from knowledge bases, i.e., SIDER and ADReCS, to include all sub-level terms and then conduct mapping between knowledge bases and EMR/FAERS. This strategy considers semantic granularity of terms in various sources and tries to obtain as many mappings as possible. **Figure 3** shows the examples of these two mapping strategies, where A refers to exact mapping and B refers to flexible mapping. For exact mapping, the term “Abdominal discomfort” is exactly mapped between SIDER/ADReCS and EMR/FAERS as this term appears in those sources. For flexible mapping, if “Skin vasculitis NOS” is detected from EMR/FAERS but only “Vasculitides” exists in the knowledge bases, we first find the lower level term of Vasculitides, i.e., “skin vasculitis NOS” and then map to the signal from EMR/FAERS. Recall, precision, and F1 measures are calculated for FAERS and EMR using the following formula (Powers, 2011). F1-measure is the harmonic mean of precision and recall (Rennie, 2004) and balances recall and precision (Yang, 1999).

3 $$Precision=\frac{Number\;ofmatchedADEs}{Numberofdis\mathrm{cov}eredADEs}$$

4 $$Recall=\frac{Number\;of\;matched\;ADEs}{Number\;of\;confirmed\;ADEs}$$

5 $$F1=\frac{2\times (Number\;of\;Matched\;ADEs)}{2\times (Number\;of\;matched\;ADEs)+number\;of\;discovered\;unmatched\;ADEs+number\;of\;undiscovered\;matched\;ADEs}$$

ADE signals detected from FAERS and/or EMR can be validated if they are also captured in SIDER/ADReCS. ADE signals detected from EMR and/or FAERS that are not validated by gold standards could very well likely be false positives but also could be potential novel ADEs, providing hypothesis for future study.

## Experiments

We tested the proposed method by detecting potential ADEs for conventional disease-modifying antirheumatic drugs (DMARDs). DMARDs include methotrexate (MTX), sulfasalazine, hydroxychloroquine and leflunomide, which are the cornerstone treatment for Rheumatoid arthritis (RA). An alternative drug choice for RA is biological agents (biologics) such as etanercept, but they are more expensive (Lee and Bae, 2016). DMARDs remain as the primary drug choice for RA treatment (Schmitz et al., 2011).

After data preparation, we extracted RA patients who have been on DMARDs for the treatment of RA. We also extracted associated records of those drugs and indication RA from AERS-DM. We then conducted signal detection for FAERS and EMR.

In validation, two files, meddra_all_label_indications.tsv.gz and meddra_all_label_se.tsv.gz from SIDER 4.1 were used in this study as gold standard for the evaluation of FAERS signals and clinical notes results. Note that not all package inserts with the same ingredient have the same adverse events. Therefore, it’s important to extract adverse events from package inserts with indications of “Rheumatoid arthritis” for drugs of interest. Using the STITCH compound identifiers of methotrexate (CID100004112), leflunomide (CID100003899), hydroxychloroquine (CID100003652) and sulfasalazine (CID105353980), package inserts with indications of “Rheumatoid arthritis” were extracted from meddra_all_label_indications.tsv.gz, and then those package inserts were used to identify recorded adverse events of these drugs.

## Results

**FIGURE 4:**
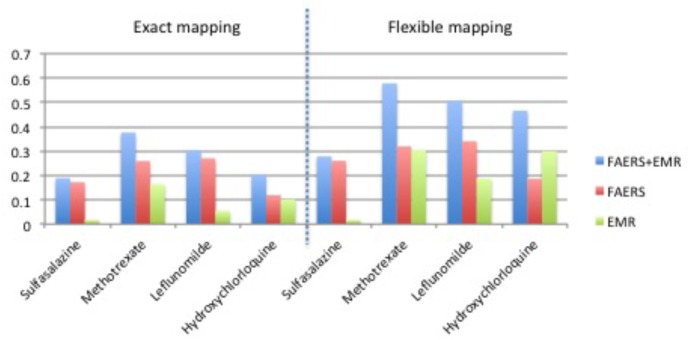
Recall in detecting ADEs using SIDER as the gold standard.

**FIGURE 5:**
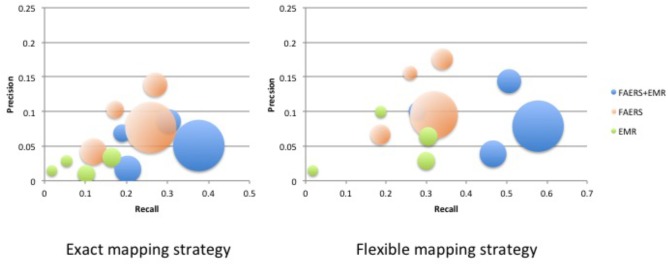
Bubble Chart of Precision, Recall and patient number for three sources (SIDER). The *X* axis denotes recall, the *Y* axis denotes precision and the bubble size denotes the number of patients.

**Table 1** shows clinical characteristics of RA patients on drugs of interest with ADE reports in FAERS and RA patients on drugs of interest in EMRs. **Supplementary Table 1** shows the recall, precision, and F1 in detecting ADEs when using ADReCS and SIDER as the gold standards. **Figure 4** shows recall (SIDER) greatly increased when combining FAERS with EMR compared with FAERS alone and EMR alone, especially for flexible mapping strategy. For example, recall for detecting ADEs associated with Methotrexate increased to 0.578 (FAERS + EMR) from 0.319 (FAERS) and 0.305 (EMR) using flexible mapping strategy. **Figure 5** (SIDER) and **Supplementary Figure S1** (ADReCS) show the three-dimensional bubble charts of precision, recall and patient number for three sources, where the bubble sizes represent the patient number. This figure demonstrates that flexible mapping strategy helps to enhance both precision and recall for three sources. **Supplementary Figure S2** shows precision (FAERS + EMR) in detecting ADEs improved using ADReCS as gold standard compared with SIDER.

**Table 1:** Clinical characteristics of patients in various sources.

Source	Gender	Sulfasalazine [report number (%)]	Methotrexate [report number (%)]	Leflunomide [report number (%)]	Hydroxychloroquine [report number (%)]
**FAERS**	Female (%)	2,312 (67.6%)	29,786 (74.2%)	5,807 (74.4%)	5,635 (79.1%)
	Male (%)	894 (26.1)	7,518 (18.7%)	1,617 (20.7%)	1,040 (14.6%)
	F:M ratio	2.6:1	4.0:1	3.6:1	5.4:1
	Total	3,420	40,161	7,820	7,123
	Female (%)	1,264 (64.8%)	4,193 (68.6%)	1,619 (70.2%)	3,985 (73.0%)
	Male (%)	687 (35.2%)	1,921 (31.4%)	686 (29.8%)	1,471 (27.0%)
	F:M ratio	1.8:1	2.2:1	2.4:1	2.0:1
	**Race**			
	American Indian/Alaskan Native	16	33	20	32
	Asian	22	56	16	56
	Black or African American	19	51	16	74
	White	1,729	5,385	2,084	4,873
	Other	34	94	38	117
	Unknown	121	467	121	278
	Choose not to disclose	10	28	10	26
	Total	1,951	6,114	2,305	5,456

**FIGURE 6:**
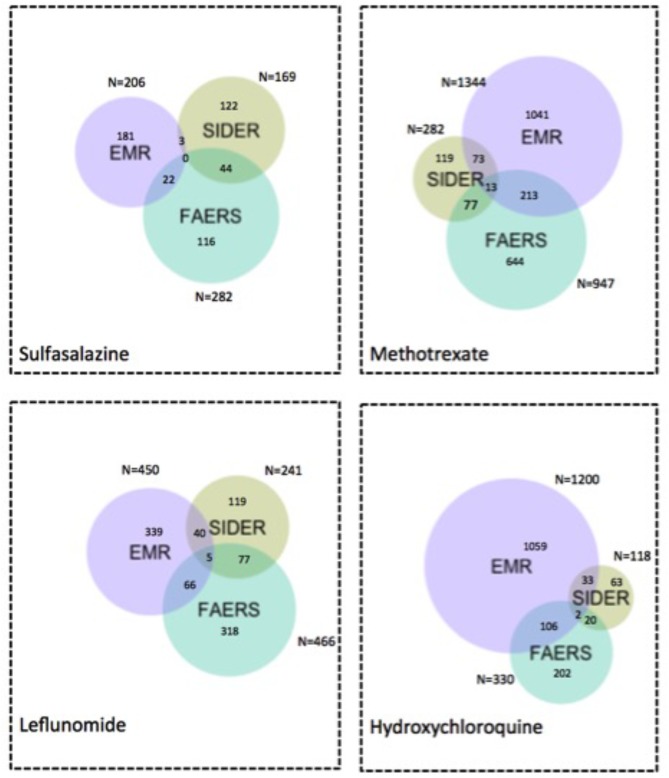
Venn diagram of ADE signals detected from FAERS and EMR and ADEs captured in SIDER using the flexible mapping strategy. Numbers indicate how many ADEs are there in each specific colored area.

**Figure 6** and **Supplementary Figure S3** show the Venn diagrams of ADE signals detected from FAERS and EMR as well as ADEs captured in SIDER, using the flexible mapping strategy and the exact mapping strategy, respectively. In **Figure 6**, the overlaps represent the typical ADEs in SIDER that are also detected from FAERS and EMR, e.g., bacterial infection, Leukocytoclastic vasculitis and sepsis for methotrexate and retinopathy for hydroxychloroquine. **Supplementary Figures S4**, **S5** show the Venn diagram of signals detected from FAERS and EMR with ADEs captured in ADReCS, using the exact mapping strategy and the flexible mapping strategy, respectively. From the Venn diagrams, it can be observed that both FAERS and EMR have a considerable number of detected signals overlapping with known ADEs captured in knowledge bases. However, there is a significant overlap between signals detected from FAERS and signals detected from EMR but not in existing knowledge bases, suggesting potential ADEs for further investigation. The top 10 potential ADEs based on the OR score are listed in **Table 2**. The disorders shown in bold letters may represent potential confounding factors due to indications. For example, diskitis is likely to be the manifestations of the underlying inflammatory arthritis for which sulfasalazine is being used. Other disorders may be potential ADEs associated with the drug. For example, methotrexate associated malignant melanoma has been confirmed in a recent study (Polesie et al., 2017). Additionally, signals detected independently by either FAERS or EMR deserve further investigation. For example, FAERS alone detected ADE signal associated with sulfasalazine, i.e., pleural effusion, which has been reported in literature to occur after sulfasalazine use (Gupta et al., 2012)but not recorded in knowledge bases. Hyperparathyroidism associated with methotrexate detected by using EMR alone has also been reported in literature (Levy et al., 2006).

**Table 2:** Top 10 potential new ADEs detected from FAERS and EMR but not recorded in ADReCS.

Drugs	ADE AAD ADE signals	OR	*P*-value	Bonferroni-corrected *P*-value
Sulfasalazine	Postoperative wound infection	8.02	3.60E-14	1.44E-12
	**Diskitis**	8.02	3.60E-14	1.44E-12
	Delayed healing	6.02	2.47E-18	9.88E-17
	Organizing pneumonia	4.01	3.82E-13	1.53E-11
	Lymph node metastasis	4.01	3.82E-13	1.53E-11
	Finger infection	4.01	3.82E-13	1.53E-11
	Staphylococcus	3.01	8.19E-05	0.003276
	Foot ulcer	2.67	0.00	0.00
	Interstitial fibrosis	2.51	6.93E-11	2.77E-09
	Septic joint	2.50	0.00	0.00
Leflunomide	Hospital acquired pneumonia	22.11	1.23E-24	4.92E-23
	Polymicrobial infection	14.04	1.23E-24	4.92E-23
	Foot cellulitis	14.04	0.01	0.40
	Septic joint	12.06	7.74E-23	3.10E-21
	Klebsiella pneumoniae	12.03	7.74E-23	3.10E-21
	Furunculosis	12.03	1.69E-20	6.76E-19
	Pseudomonas pneumonia	10.02	1.72E-26	6.88E-25
	**Hip dislocation**	9.03	7.05E-18	2.82E-16
	**Subtrochanteric fracture**	8.01	7.05E-18	2.82E-16
	Perforated gastric ulcer	8.01	0.02	0.80
Methotrexate	Lymphoproliferative disorder	34.09	1.05E-12	4.20E-11
	**Shoulder fracture**	16.02	2.19E-32	8.76E-31
	Malignant melanoma *in situ*	16.02	2.19E-32	8.76E-31
	Bacterial pneumonia	16.02	2.19E-32	8.76E-31
	Hospital acquired pneumonia	14.03	5.39E-07	2.16E-05
	Varicella zoster	14.02	2.05E-30	8.20E-29
	B cell lymphoma	12.04	0.01	0.40
	**Osteoporotic fracture**	12.02	0.00	0.00
	Chest congestion	12.02	0.00	0.00
	**Wrist injury**	12.01	1.47E-28	5.88E-27
Hydroxychloroquine	Postoperative infection	12.01	7.51E-40	3.00E-38
	**Rheumatoid arthropathy**	8.01	5.13E-43	2.05E-41
	Metastatic carcinoma	8.01	5.13E-43	2.05E-41
	**Hip dislocation**	8.01	5.13E-43	2.05E-41
	SLE flare	8.01	5.47E-34	2.19E-32
	**Lumbar fracture**	8.01	5.47E-34	2.19E-32
	Injection site reactions	8.01	5.47E-34	2.19E-32
	Ghon complex	8.01	5.47E-34	2.19E-32
	Upper extremity edema	5.00	2.30E-35	9.20E-34
	**Shoulder dislocation**	5.00	2.30E-35	9.20E-34

## Discussion

Drug safety surveillance in clinical practice is important for each drug after introduction on the market. In this study we propose a method for ADE detection from EMR and FAERS and used DMARDs for the treatment of RA as the example case. Some detected ADEs can be confirmed by knowledge bases, and some are potentially new ADEs detected from EHR and/or FAERS but not in existing knowledge bases, warranting further studies for validation.

Researchers have tried to build comprehensive ADE knowledge bases but currently there are no well established ones. One of reasons lies in the fact that post-marketing surveillance is an ongoing process. Though SIDER is included in ADReCS, the comparison of SIDER and ADReCS strengthens our method as some false positives detected from SIDER are true ADEs captured in ADReCS.

The findings from **Supplementary Table S1** demonstrated that flexible mapping strategy helped to improve recall, precision and F1 on the whole. One possible reason maybe because EMR clinical narratives contain dense ADE signals in various semantic granularities where exact mapping strategy may miss some mappings. ADE signals detected from EMR and/or FAERS that are not validated by gold standards could very well likely be false positives but also could be potential novel ADEs.

In our previous study, we demonstrated the feasibility of EMR in validating potential ADEs from FAERS (Wang et al., 2017b). The present study focuses on a method for ADE detection from EMR and FAERS. Our method offers multiple contributions to the community. First, we use NLP techniques to extract treatment outcome information captured in unstructured text and standardize the information using UMLS codes which enables us to detect potential ADEs not captured in structured EMR data and at the same time, allows us to adopt the flexible mapping strategy to combine signals detected from diverse data sources. Secondly, our method adopts case-crossover design with drug use as exposure. This method can automatically eliminate many underlying confounding factors and false positive signals, especially after the incorporation of temporal information of treatment outcomes and drug uses.

A previous study investigated a large-scale signal boosting approach combining FAERS and MEDLINE articles (Xu and Wang, 2014), where pairs appearing in both FAERS and MEDLINE sentences had the highest precision of 0.140 compared to 0.025 (FAERS) and 0.111 (FAERS + abstract), recall varied from 0.507 (FAERS), 0.138 (FAERS and sentences) to 0.234 (FAERS + abstract), and F1 varied from 0.045 (FAERS), 0.139 (FAERS and sentences) to 0.151 (FAERS + abstract). The study used SIDER as the gold standard, and the findings represented the performance level for large-scale databases like FAERS and MEDLINE as roughly from 0.025 to 0.507. Our results showed similar performances using another large-scale database, i.e., EMR.

Various sources of ADEs could exert different effects with unique advantages. FAERS includes voluntary spontaneous reports that may not capture the true incidence information. However, it contains millions of ADE-relevant records. Meanwhile, as a real world data source with the capability to yield valuable insights, EMR contains abundant information on treatment outcome and drug exposure. Therefore, a higher number of false positives by combining EMR and FAERS implies more potential novel ADEs detected. In addition, ADE signals detected by both FAERS and EMR can be prioritized for follow-up epidemiological or laboratory studies.

Limitations of our study include the following. First, our experiment used EMR data from a single institution. Secondly, the design of the case crossover studies did not exclude complex drug exposure situation and drug–drug interactions, and the carry over effects of previous drugs during the “control” period and other time-varying confounders that could result in ADE such as new comorbidities of patients. Additionally, the semantic granularity difference across diverse data sources has not been fully addressed even through the flexible mapping strategy. We plan to tackle those limitations by using EMR data from multiple institutions and investigate strategies to exclude complex drug exposure situation. Also, we can adopt semantic similarity to address the semantic granularity difference issue.

## Conclusion

We proposed a method for detecting potential ADEs associated with drugs by combining two data sources: FAERS and EMR, and evaluated their performances using two ADE knowledge bases: ADReCS and SIDER. The use of NLP and the innovative adoption of case-crossover study design enable us to detected signals from large-scale EMR data. Findings showed that Recall greatly increased when combining FAERS with EMR compared with FAERS alone and EMR alone, especially for flexible mapping strategy. Precision (FAERS + EMR) in detecting ADEs improved using ADReCS as gold standard compared with SIDER. In addition, signals detected from EMR have considerably overlapped with signals detected from FAERS or ADE knowledge bases, implying the importance of EMR for pharmacovigilance. ADE signals detected from EMR and/or FAERS but not in existing knowledge bases provide hypothesis for future study.

## Author Contributions

All co-authors are justifiably credited with authorship, according to the authorship criteria. Final approval is given by each co-author. LW: conception, design, development, data collection, analysis of data, interpretation of results, drafting and revision of manuscript. MR-M: design, data collection, analysis of data, interpretation of results and revision of manuscript. ZJ, SL, KL, SM, FS, and YW: analysis of data. LY: revision of manuscript. JD: interpretation of results and critical revision of manuscript. HL: conception, design, development, data collection, analysis of data, interpretation of results and critical revision of manuscript.

## Conflict of Interest Statement

The authors declare that the research was conducted in the absence of any commercial or financial relationships that could be construed as a potential conflict of interest.

## Footnotes

1 http://informatics.mayo.edu/adepedia/index.php/Download

## Supplementary Material

FIGURE S1 Bubble chart of precision, recall and patient number for three sources (ADReCS). The *X* axis denotes recall, the *Y* axis denotes precision and the bubble size denotes the number of patients.

FIGURE S2 Precision of FAERS + EMR in detecting ADEs when using ADReCS and SIDER as the gold standards.

FIGURE S2 Venn diagram of ADE signals detected from FAERS and EMR and ADEs captured in SIDER using the exact mapping strategy. Numbers indicate how many ADEs are there in each specific colored area.

FIGURE S4 Venn diagram of ADE signals detected from FAERS and EMR and ADEs captured in ADReCS using the exact mapping strategy. Numbers indicate how many ADEs are there in each specific colored area.

FIGURE S5 Venn diagram of ADE signals detected from FAERS and EMR and ADEs captured in ADReCS using the flexible mapping strategy. Numbers indicate how many ADEs are there in each specific colored area.

TABLE S1 Performance of FAERS, EMR and FAERS + EMR in detecting ADEs when using ADReCS and SIDER as the gold standards.
