# Supplementary figures and images for: Detecting Pharmacovigilance Signals Combining Electronic Medical Records With Spontaneous Reports: A Case Study of Conventional Disease-Modifying Antirheumatic Drugs for Rheumatoid Arthritis
Source: Front Pharmacol. 2018 Aug 7;9:875. doi: 10.3389/fphar.2018.00875 (PMC6090179; doi:10.3389/fphar.2018.00875)

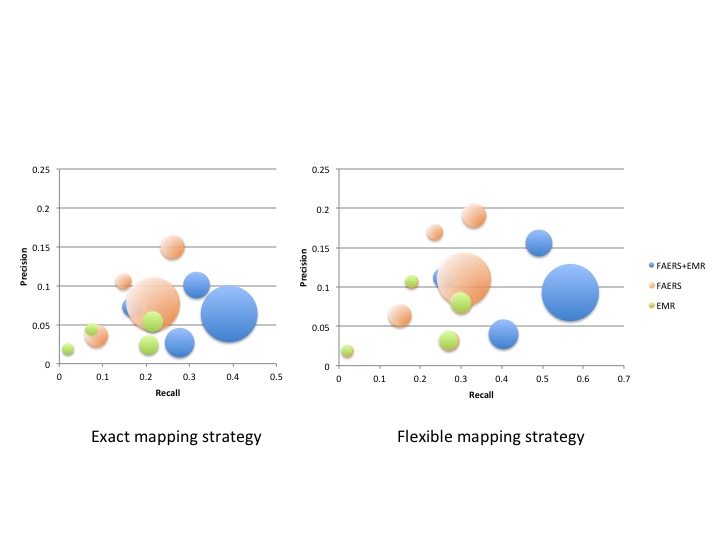

Supplement: FIGURE S1 — Bubble chart of precision, recall and patient number for three sources (ADReCS). The X axis denotes recall, the Y axis denotes precision and the bubble size denotes the number of patients. [file Image_1.JPEG]

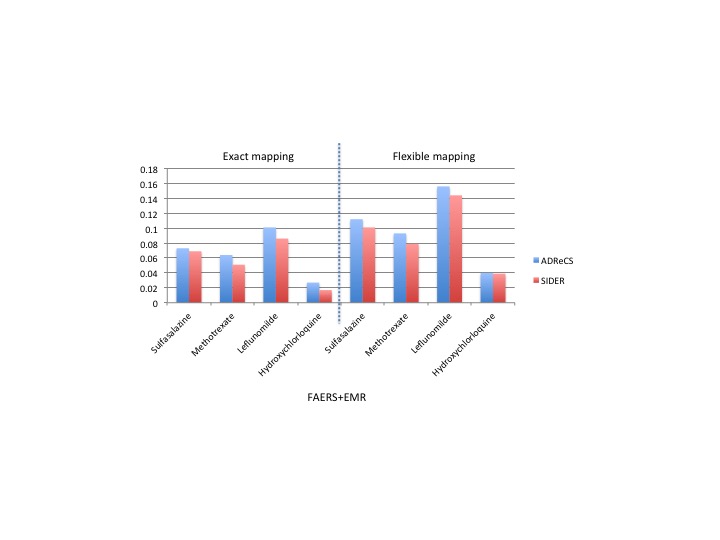

Supplement: FIGURE S2 — Precision of FAERS + EMR in detecting ADEs when using ADReCS and SIDER as the gold standards. [file Image_2.JPEG]

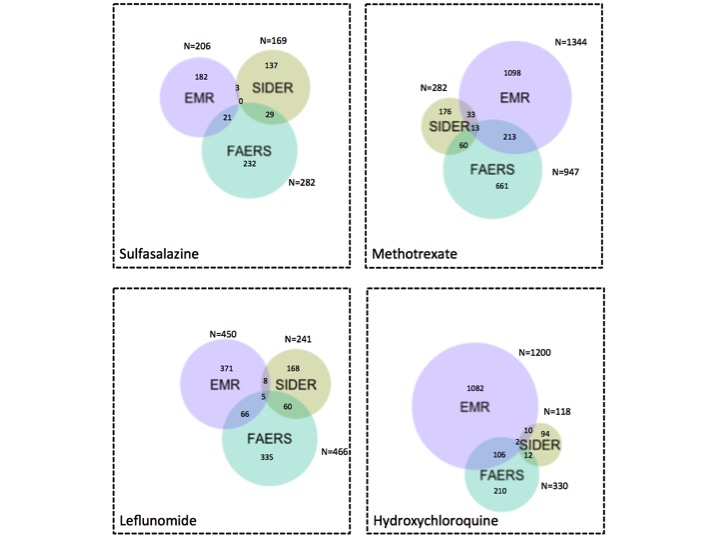

Supplement: FIGURE S2 — Venn diagram of ADE signals detected from FAERS and EMR and ADEs captured in SIDER using the exact mapping strategy. Numbers indicate how many ADEs are there in each specific colored area. [file Image_3.JPEG]

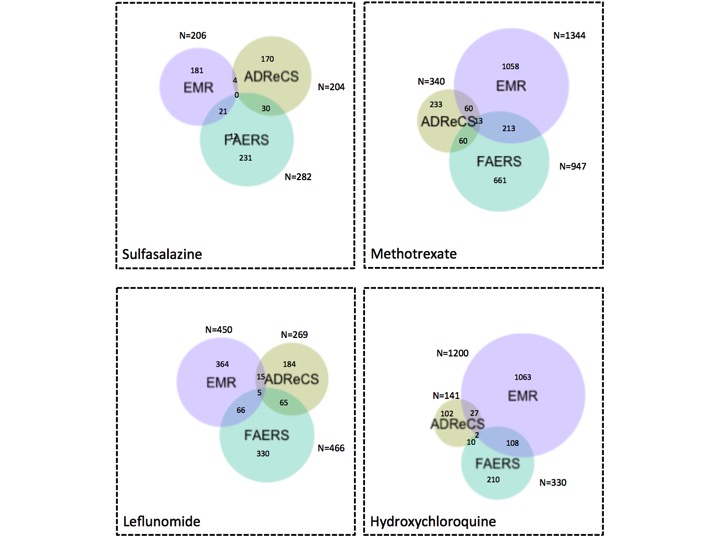

Supplement: FIGURE S4 — Venn diagram of ADE signals detected from FAERS and EMR and ADEs captured in ADReCS using the exact mapping strategy. Numbers indicate how many ADEs are there in each specific colored area. [file Image_4.JPEG]

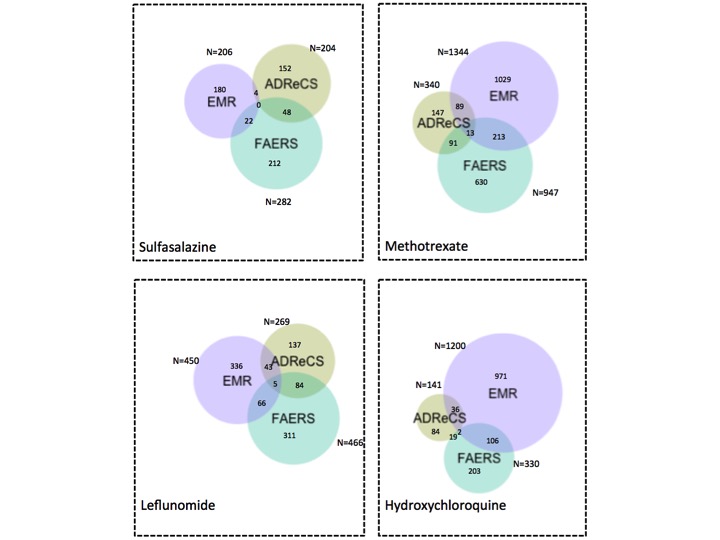

Supplement: FIGURE S5 — Venn diagram of ADE signals detected from FAERS and EMR and ADEs captured in ADReCS using the flexible mapping strategy. Numbers indicate how many ADEs are there in each specific colored area. [file Image_5.JPEG]
