# Supplementary material for: Detecting Pharmacovigilance Signals Combining Electronic Medical Records With Spontaneous Reports: A Case Study of Conventional Disease-Modifying Antirheumatic Drugs for Rheumatoid Arthritis
Source: Front Pharmacol. 2018 Aug 7;9:875. doi: 10.3389/fphar.2018.00875 (PMC6090179; doi:10.3389/fphar.2018.00875)
Supplement: TABLE S1 — Performance of FAERS, EMR and FAERS + EMR in detecting ADEs when using ADReCS and SIDER as the gold standards. [file Table_1.DOCX]

Supplemental Table 1**.** Performance of FAERS, EMR and FAERS+EMR in detecting ADEs when using ADReCS and SIDER as the gold standards

| **Performance in various sources** | | **Sulfasalazine** | | **Methotrexate** | | **Leflunomide** | | **Hydroxychloroquine** | |
| --- | --- | --- | --- | --- | --- | --- | --- | --- | --- |
|  | | Exact | Flexible | Exact | Flexible | Exact | Flexible | Exact | Flexible |
| Recall (ADReCS) | FAERS+EMR | 0.167 | 0.255 | 0.391 | 0.568 | 0.316 | 0.491 | 0.277 | 0.404 |
|  | FAERS | 0.147 | 0.235 | 0.215 | 0.306 | 0.260 | 0.331 | 0.085 | 0.149 |
|  | EMR | 0.020 | 0.020 | 0.215 | 0.300 | 0.074 | 0.178 | 0.206 | 0.270 |
| Precision  (ADReCS) | FAERS+EMR | 0.073 | 0.112 | 0.064 | 0.093 | 0.101 | 0.156 | 0.027 | 0.040 |
|  | FAERS | 0.106 | 0.170 | 0.077 | 0.110 | 0.150 | 0.191 | 0.036 | 0.064 |
|  | EMR | 0.019 | 0.019 | 0.054 | 0.08 | 0.044 | 0.107 | 0.024 | 0.032 |
| F1  (ADReCS) | FAERS+EMR | 0.101 | 0.155 | 0.111 | 0.160 | 0.153 | 0.237 | 0.050 | 0.073 |
|  | FAERS | 0.123 | 0.200 | 0.113 | 0.162 | 0.190 | 0.242 | 0.051 | 0.089 |
|  | EMR | 0.020 | 0.020 | 0.087 | 0.121 | 0.056 | 0.134 | 0.043 | 0.057 |
| Recall (SIDER) | FAERS+EMR | 0.189 | 0.278 | 0.376 | 0.578 | 0.303 | 0.506 | 0.203 | 0.466 |
|  | FAERS | 0.172 | 0.260 | 0.259 | 0.319 | 0.270 | 0.340 | 0.119 | 0.186 |
|  | EMR | 0.018 | 0.018 | 0.1638 | 0.305 | 0.054 | 0.187 | 0.102 | 0.300 |
| Precision (SIDER) | FAERS+EMR | 0.069 | 0.101 | 0.051 | 0.079 | 0.086 | 0.144 | 0.017 | 0.039 |
|  | FAERS | 0.103 | 0.156 | 0.077 | 0.095 | 0.139 | 0.176 | 0.042 | 0.067 |
|  | EMR | 0.015 | 0.015 | 0.034 | 0.064 | 0.029 | 0.100 | 0.0100 | 0.029 |
| F1 (SIDER) | FAERS+EMR | 0.100 | 0.148 | 0.090 | 0.139 | 0.134 | 0.225 | 0.031 | 0.071 |
|  | FAERS | 0.129 | 0.195 | 0.119 | 0.146 | 0.184 | 0.232 | 0.063 | 0.098 |
|  | EMR | 0.016 | 0.016 | 0.057 | 0.106 | 0.038 | 0.130 | 0.018 | 0.053 |
